# Supplementary material for: Bombyx Vasa sequesters transposon mRNAs in nuage via phase separation requiring RNA binding and self-association
Source: Nat Commun. 2023 Apr 7;14:1942. doi: 10.1038/s41467-023-37634-2 (PMC10081994; doi:10.1038/s41467-023-37634-2)
Supplement: Supplementary file 1 — Supplementary Information [file 41467_2023_37634_MOESM1_ESM.pdf]

Supplementary Information

Bombyx Vasa sequesters transposon mRNAs in nuage via phase separation requiring RNA binding and self-association

Yamazaki et al.

Table of contents

Supplementary Figures

Supplementary Figure.1 Unique trait of each domain in BmVasa.

Supplementary Figure 2 LLPS of recombinant BmVasa proteins and behavior of BmVasa RK mutant in BmN4 cells.

Supplementary Figure 3 The droplets of BmVasa WT and R470A, and FRAP analysis of the droplets of BmVasa WT and E339Q.

Supplementary Figure 4 N-IDR sequence alignment.

Supplementary Figure 5 Bioinformatic analysis of BmVasa FAST-iCLIP reads.

a

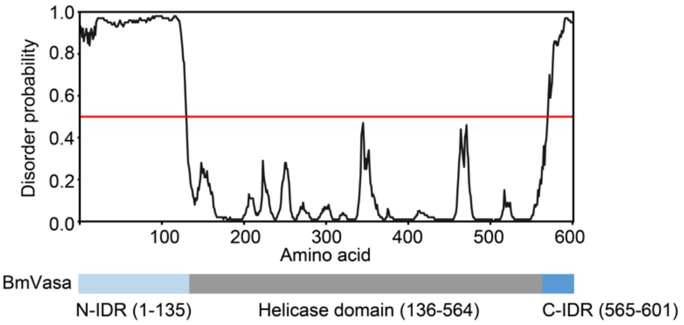


```
MDDDWDDSCEAVVVPPPPLQNHDSVDEGHSLSRGRGFPSF
NEDDEKENGYGERRGRGGRGGGRGRGGRGGRGGSREQHSD     N-IDR (1-135)
YETNGDHDDRGRGERGRGRGRGGRGGGRGGGGGGGDRNDY
EDNEIGENGETKKPVTYVPPEPTNDETEIFSSTISSGINF
DKFDHIAVKVSGENPPRPIESFETANLRKYVLDNVLKAGY
RKPTPIQKNAIPIIMSGRDLMGCAQTGSGKTAAFLVPIIN     Helicase domain
MLLQDPKDLISENGCAQPQVIIVSPTRELTLQIFNEARKF     (136-564)
SYGSVLKVAVAYGGTAVRHQGDNIARGCHILVATPGRLHD
FVERNRVSFGSVRFVVLDEADRMLDMGFMPSIEKMMLHPT     DEAD (338-341)
MVETTKRQTLMFSATFPEDIQHLAGRFLNNYLFVAVGIVG
GASTDVEQIFIEVTKYEKRNSLKQLIEENDGKRILVFVET
KRNADFIAAMLSEQQLLTSSIHGDRMQREREEALQNFKSG     Arg470
KHCILVATAVAARGLDIKNVDIVVNYDLPKSIDEYVHRIG
RTGRVGNRGKAVSFYDSDQDLALVADLSKILRQADQSVPD
FLKGGGTATFKGNKYGGSDVRNFNNANTAVVDQGQEPEEE     C-IDR (565-601)
W
```

b

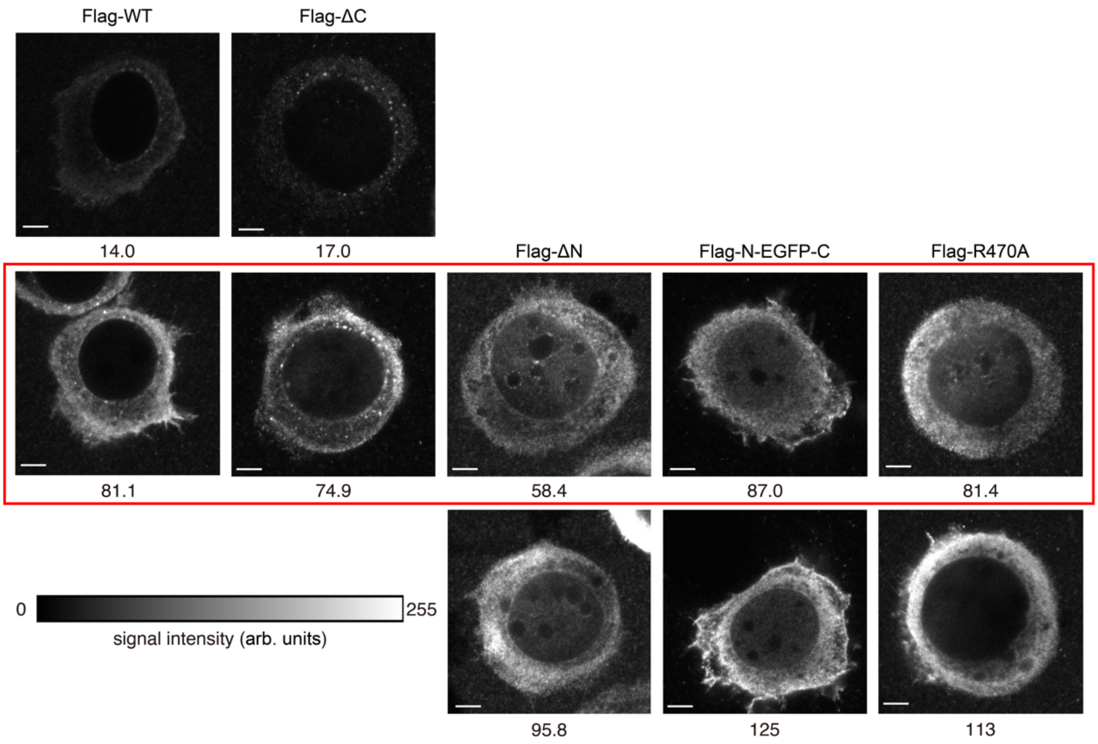

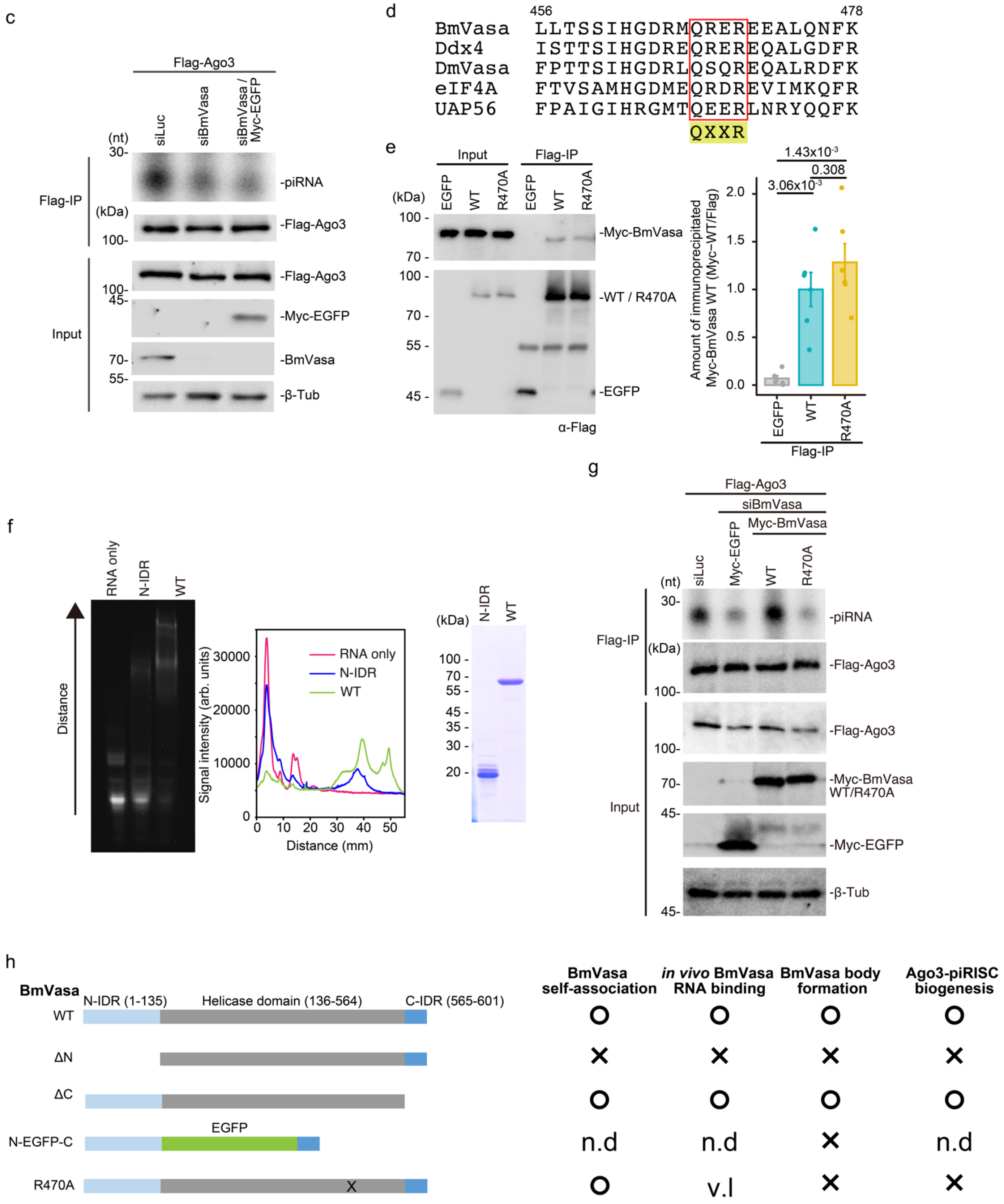
c
Flag-Ago3
siLuc
siBmVasa
siBmVasa / Myc-EGFP
(nt)
30-
-piRNA
Flag-IP
(kDa)
-Flag-Ago3
100-
-Flag-Ago3
100-
45-
-Myc-EGFP
Input
70-
-BmVasa
55-
-β-Tub
d
456
478
BmVasa LLTSSIHGDRMQREREEALQNFK
Ddx4 ISTTSIHGDREQREREQALGDFR
DmVasa FPTTSIHGDRLQSQREQALRDFK
eIF4A FTVSAMHGDMEQRDREVIMKQFR
UAP56 FPAIGIHRGMTQEERLNRYQQFK
QXXR
e
Input
Flag-IP
(kDa)
EGFP
WT
R470A
EGFP
WT
R470A
100 -
-Myc-BmVasa
70 -
100 -
-WT / R470A
70 -
55 -
45 -
-EGFP
α-Flag
1.43x10-3
0.308
3.06x10-3
Amount of immunoprecipitated Myc-BmVasa WT (Myc-WT/Flag)
2.0
1.5
1.0
0.5
0.0
EGFP
WT
R470A
Flag-IP
f
RNA only
N-IDR
WT
Distance
Signal intensity (arb. units)
30000
20000
10000
0
RNA only
N-IDR
WT
0
10
20
30
40
50
Distance (mm)
(kDa)
N-IDR
WT
100 -
70 -
55 -
45 -
35 -
30 -
20 -
g
Flag-Ago3
siBmVasa
Myc-BmVasa
siLuc
Myc-EGFP
WT
R470A
(nt)
30-
-piRNA
Flag-IP
(kDa)
-Flag-Ago3
100-
-Flag-Ago3
100-
70-
-Myc-BmVasa WT/R470A
Input
45-
-Myc-EGFP
-β-Tub
45-
h
BmVasa
N-IDR (1-135)
Helicase domain (136-564)
C-IDR (565-601)
WT
ΔN
ΔC
EGFP
N-EGFP-C
R470A
X
BmVasa self-association
in vivo BmVasa RNA binding
BmVasa body formation
Ago3-piRISC biogenesis
WT: O O O O
ΔN: × × × ×
ΔC: O O O O
N-EGFP-C: n.d n.d × n.d
R470A: O v.l × ×

Supplementary Figure 1. Unique trait of each domain in BmVasa. (a) Disorder probability shows the N-IDR (residues 1–135) and the C-IDR (residues 565–601) in BmVasa. Red line on the disorder plots represents disorder probability = 0.5. The domain structure and amino acid sequence of BmVasa are also indicated. Light blue: N-IDR. Dark blue: C-IDR. Yellow: DEAD box. Red: Arg470. The RNA helicase domain: residues 136–564. **(b)** Subcellular localization of Flag-BmVasa WT (Flag-WT), Flag-BmVasa ΔC mutant (Flag-ΔC), Flag-BmVasa ΔN mutant (Flag-ΔN), Flag-BmVasa N-EGFP-C mutant (Flag-N-EGFP-C), and Flag-BmVasa R470A mutant (Flag-R470A) in BmN4 cells (gray) at various expression levels. Grayscale bar indicates signal intensity. Numerical value below the image is the mean signal intensity (a.u) in cytoplasm. Scale bar: 5 μm. **(c)** piRNAs loaded onto Flag-Ago3 in BmVasa-depleted BmN4 cells (BmVasa KD). siLuc (Control) and Myc-EGFP were used as negative controls. Flag-IP, upper: ^{32}P-labeled piRNAs bound to Flag-Ago3. Flag-IP, lower: western blotting shows Flag-Ago3 in the immunoprecipitates. Input, from the top: Western blotting shows the levels of Flag-Ago3, Myc-EGFP, endogenous BmVasa, and β-Tubulin (β-Tub; a loading control) in input. **(d)** Conservation of Q-X-X-R and its surroundings in BmVasa (residues 456–478), Ddx4, DmVasa, and eIF4A of *Drosophila melanogaster* (UniProt: Q02748), and UAP56 of *Drosophila melanogaster* (UniProt: Q27268). The red box shows Q-X-X-R in each protein. **(e)** Self-association of WT BmVasa and the R470A mutant. Anti-Flag antibody was used for immunoprecipitation and western blotting (left). Anti-Myc antibody was used for western blotting (upper gel). Flag-EGFP was used as a negative control (lower gel). Myc-BmVasa signal was divided by Flag-tagged protein signal and plotted (right) (Error bar: standard error). Two-tailed Welch's t-test was performed and p values are indicated on the graph (n = 6). **(f)** Electrophoretic mobility shift assay was performed with BmVasa WT, N-IDR and 50 nt ssRNA. Protein/RNA mixture was subjected to electrophoresis with non-denaturing polyacrylamide gel. The gel was stained with SYBR Gold to visualize RNA and is shown on the left. Quantified SYBR Gold signal along with the arrow was shown in the middle. BmVasa WT and N-IDR CBB-stained (SDS-PAGE) are shown on the right. **(g)** piRNAs loaded onto Flag-Ago3 in BmVasa-depleted BmN4 cells (siBmVasa). Prior to immunoprecipitation, Myc-BmVasa WT and Myc-BmVasa R470A were individually expressed. Myc-EGFP was used as a negative control. Flag-IP, upper: ^{32}P-labeled piRNAs bound to Flag-Ago3. Flag-IP, lower: western blotting shows Flag-Ago3 in the immunoprecipitates. Input, from the top: Western blotting shows Flag-Ago3, Myc-BmVasa WT, Myc-BmVasa R470A, Myc-EGFP, and β-Tubulin (β-Tub; a loading control) in input. **(h)** Summary of functional characteristics of each BmVasa mutant analyzed in this study and WT BmVasa. The domain structures of the proteins are also shown. v.l: very low. n.d: not determined. Source data are provided as a Source Data file.

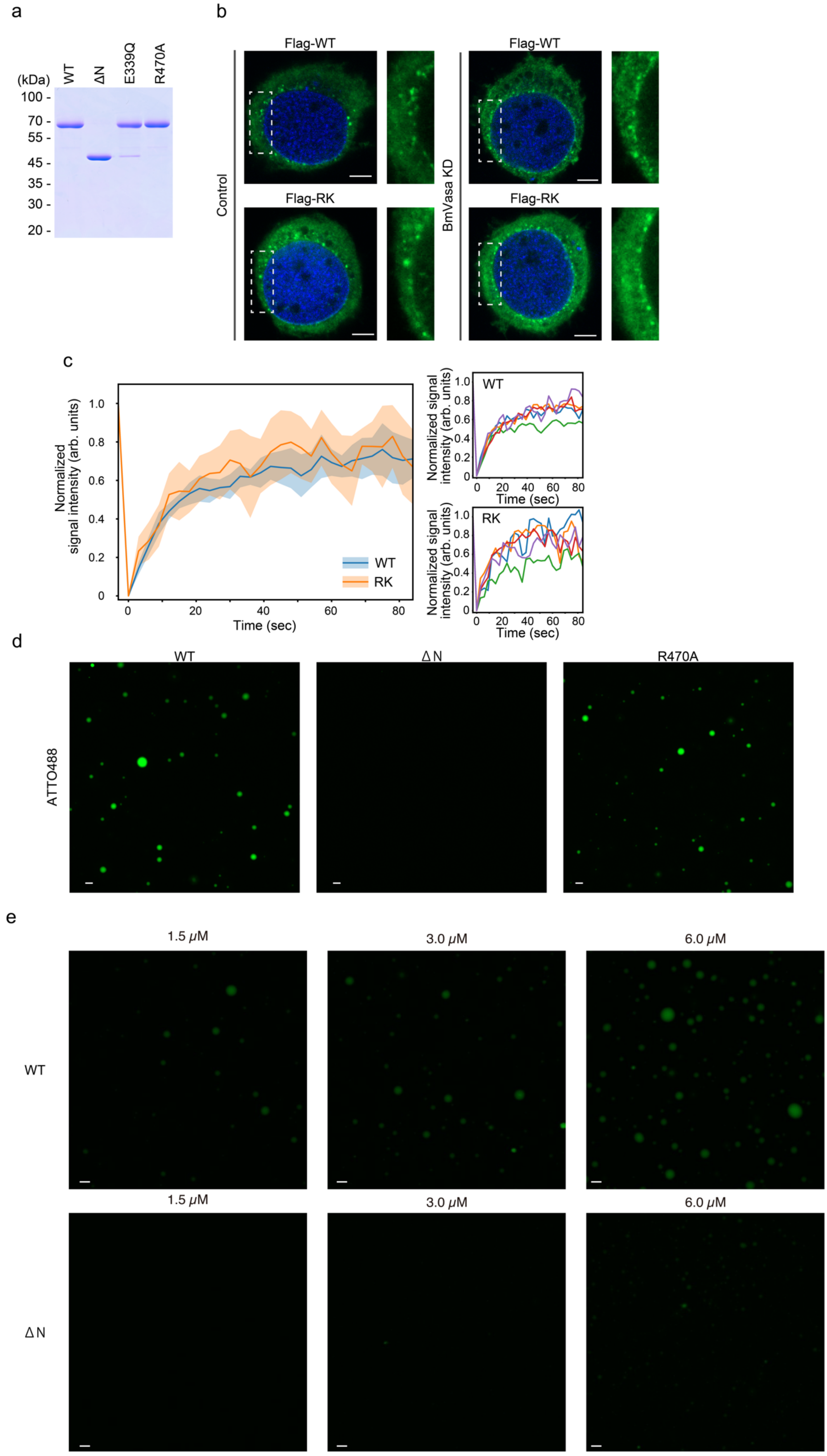
a
(kDa)
WT
ΔN
E339Q
R470A
100 -
70 -
55 -
45 -
35 -
30 -
20 -
b
Control
BmVasa KD
Flag-WT
Flag-RK
c
Normalized signal intensity (arb. units)
Time (sec)
WT
RK
d
ATTO488
WT
Δ N
R470A
e
1.5 μM
3.0 μM
6.0 μM
WT
Δ N

Supplementary Figure 2. LLPS of recombinant BmVasa proteins and behavior of BmVasa RK mutant in BmN4 cells. (a) CBB-stained protein gel shows the purity of BmVasa WT and its mutants, ΔN, E339Q, and R470A, employed in the *in vitro* droplet formation assay. **(b)** Subcellular localization of Flag-BmVasa WT and RK mutant (Flag-RK) (green) in control BmN4 cells (Control) and cells lacking endogenous BmVasa (BmVasa KD). DAPI (blue): nuclei. Scale bar: 5 μm. Enlarged images of the insets indicated with white dotted lines are shown on the right. **(c)** FRAP analysis of the EGFP-BmVasa WT and RK expressed in BmN4 and localized in Vasa bodies was performed. Mean (line) and standard deviation (shade) are shown (left) (n = 5). Individual data are plotted on the right. **(d)** Uncropped images of Fig. 2g. Scale bar: 5 μm. **(e)** *In vitro* droplet formation assay in the presence of 15% PEG 6000 was performed with different concentrations of BmVasa WT and ΔN. Scale bar: 5 μm. Source data are provided as a Source Data file.

Supplementary Figure 3

Yamazaki et al.

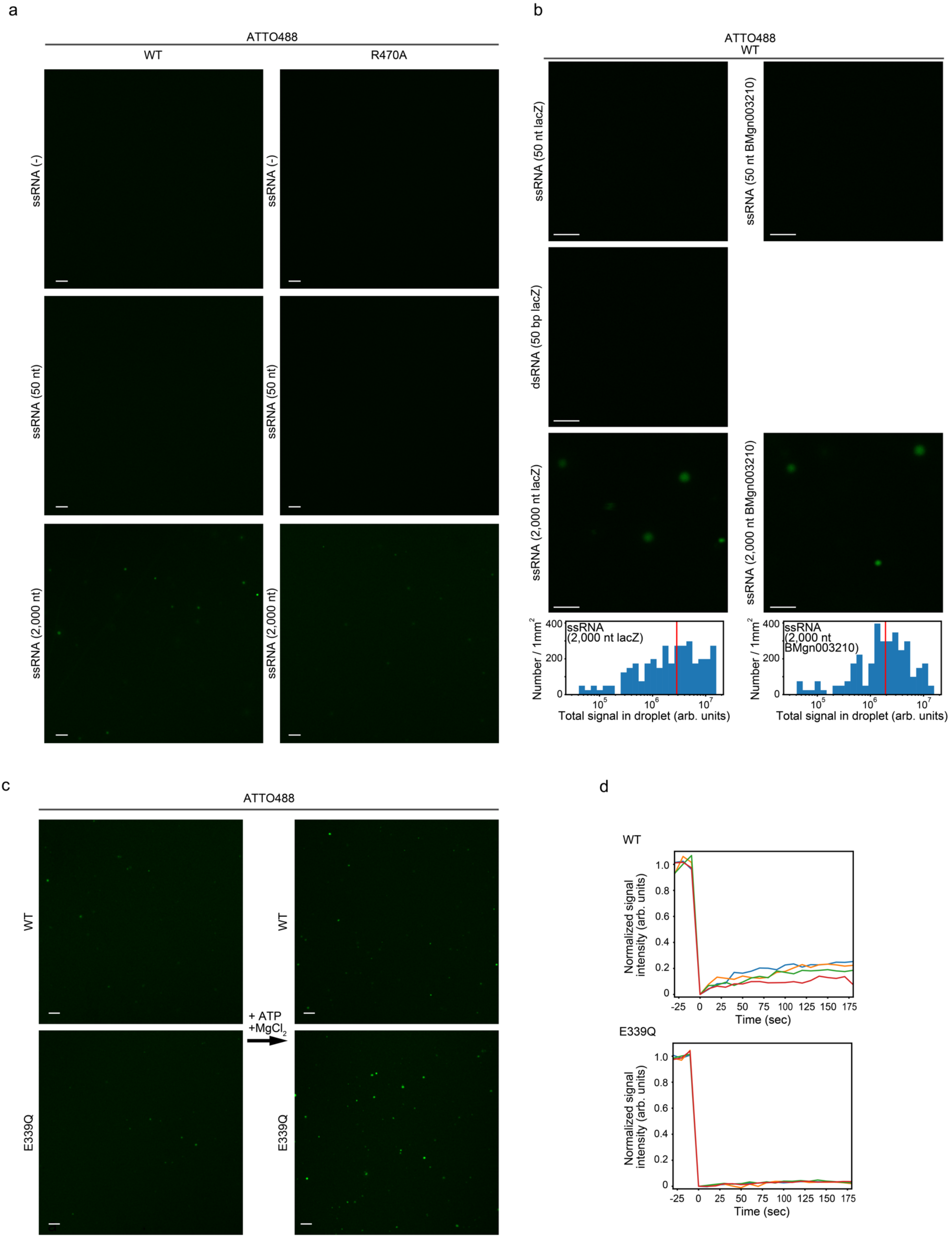

Supplementary Figure 3. The droplets of BmVasa WT and R470A, and FRAP analysis of the droplets of BmVasa WT and E339Q. (a) Uncropped images of Fig. 3a. Scale bar: 5 μm. **(b)** *In vitro* droplet formation assay of ATTO488-labeled recombinant BmVasa WT (1.5 μM) in the presence of 5% PEG 6000 and 50 nt ssRNA (derived from lacZ or BMgn003210), 50 bp dsRNA (derived from lacZ) or 2,000 nt ssRNA (derived from lacZ or BMgn003210). Only the 2,000 nt ssRNA promoted droplet formation. Histograms of total ATTO488 signal intensity of a droplet in the presence of 2,000 nt ssRNAs are shown. Red line indicates the median; the difference between 2,000 nt ssRNA derived from lacZ and BMgn003210 was small for clarity. ($p = 0.0406$ by two-tailed Mann–Whitney U test). Scale bar: 5 μm. **(c)** Uncropped images of Fig. 3b. Scale bar: 5 μm. **(d)** FRAP analysis of the droplets of BmVasa WT and E339Q in the presence of 2,000-nt-long ssRNAs, ATP, and $MgCl_2$. Individual data ($n = 4$) of Fig. 3c are shown. Source data are provided as a Source Data file.

```
BmVasa    1 MDDDWDDSCEAVVVPPPPLQNHDSVDEGHSLSRG-RGFPSFNEDDEKENG
DmVasa    1 MSDDWDDE---------PI-----VD-----TRGARGGDWSDDEDTAKSF

BmVasa   50 YGERRGRG-------------------GR-GGGRGRGGRGGRGGSREQHS
DmVasa   32 SGEAEGDGVGGSGGEGGGYQGGNRDVFGRIGGGRGGGAGGYRGGNR----

BmVasa   80 DYETNGDHDDRGRGER-----------GRGRGRGGRGGGRGGGGG-----
DmVasa   78 --DGGGFHGGRREGERDFRGGEGGFRGGQGGSRGGQGGSRGGQGGFRGGE

BmVasa  114 -------------------------GGDRNDYEDNEI--GENGE------
DmVasa  126 GGFRGRLYENEDGDERRGRLDREERGGERRGRLDREERGGERGERGDGGF

BmVasa  131 --------------------TKKPVTYVPPEPTNDETEIFSSTISSGINF
DmVasa  176 ARRRRNEDDINNNNNIVEDVERKREFYIPPEPSNDAIEIFSSGIASGIHF
```

Supplementary Figure 4. N-IDR sequence alignment. The sequences of BmVasa and DmVasa were subjected to pairwise sequence alignment. Amino acids 1–160 of BmVasa and 1–225 of DmVasa are shown. Blue and orange highlights indicate N-IDR and conserved amino acid, respectively.

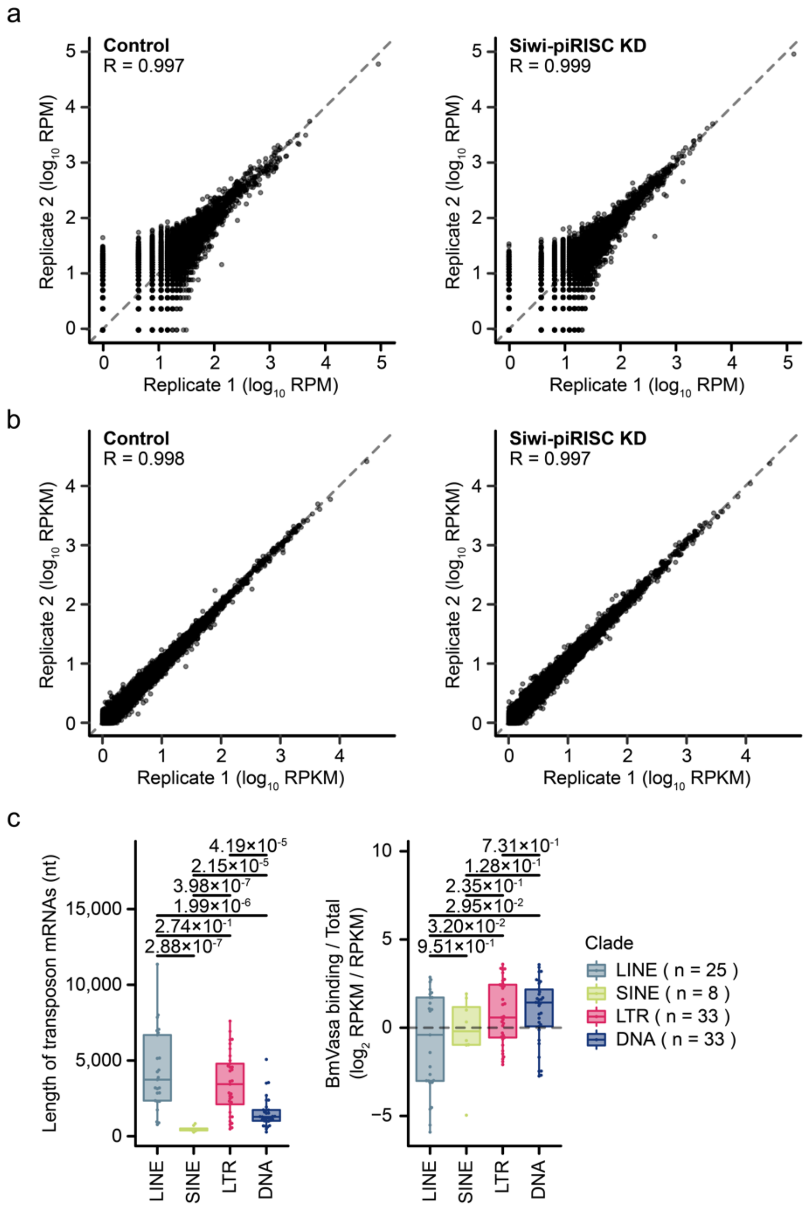

Supplementary Figure 5. Bioinformatic analysis of BmVasa FAST-iCLIP reads. (a) Scatter plots show RPM values of the BmVasa FAST-iCLIP library (BmVasa binding) for mRNAs in two replicates produced in control BmN4 cells (left) and cells lacking Spn-E (Siwi-piRISC KD; right). R is Pearson's correlation coefficient. **(b)** Scatter plots show the expression levels of mRNAs in two replicates produced in control BmN4 cells (left) and cells lacking Siwi (Siwi-piRISC KD; right). R is Pearson's correlation coefficient. **(c)** Box plots show the length distribution (left) and the enrichment value (BmVasa binding relative to Total; right) of transposon mRNAs with the annotation of clade. Two-tailed Mann–Whitney U test was performed and p values are indicated in the box plots. Center line: median. Box limits: upper and lower quartiles. Whiskers: 1.5 × interquartile range. Source data are provided as a Source Data file.

Supplementary Table 1. Sequences of oligonucleotides

Experiment	Name	Sequence*
Plasmid Construction	3xFLAG-BmVasa ΔN Forward (vector)	ACGCGTACCGGTCATCATCACCATCAC
	3xFLAG-BmVasa ΔN Reverse (vector)	GAATTCGATATCCTTGTCATCGTCATC
	3xFLAG-BmVasa ΔN Forward (insert)	AAGGATATCGAATTCACCTATGTGCCTCCAGAACCA
	3xFLAG-BmVasa ΔN Reverse (insert)	ATGACCGGTACGCGTCTACCATTCTTCTTCAGGTTC
	3xFLAG-BmVasa ΔC Forward (vector)	ACGCGTACCGGTCATCATCACCATCAC
	3xFLAG-BmVasa ΔC Reverse (vector)	GAATTCGATATCCTTGTCATCGTCATC
	3xFLAG-BmVasa ΔC Forward (insert)	AAGGATATCGAATTCATGGATGATGACTGGGATGAT
	3xFLAG-BmVasa ΔC Reverse (insert)	ATGACCGGTACGCGTCTACTTTAAGAAGTCTGGGAC
	3xFLAG-BmVasa N-EGFP-C Forward (vector)	GGAGGTGGCACTGCTACATTCAAG
	3xFLAG-BmVasa N-EGFP-C Reverse (vector)	TACCGGCTTCTTCGTTTCCCCGTT
	3xFLAG-BmVasa N-EGFP-C Forward (insert)	ACGAAGAAGCCGGTAATGGTGAGCAAGGGCGAGGAG
	3xFLAG-BmVasa N-EGFP-C Reverse (insert)	AGCAGTGCCACCTCCCTTGTACAGCTCGTCCATGCC
	3xFLAG-BmVasa RK Forward (vector)	ACCTATGTGCCTCCAGAACCAAC
	3xFLAG-BmVasa RK Reverse (vector)	CATCCATGAATTCGATATCCTTGTCATCG
	3xFLAG-BmVasa RK Forward (insert)	TCGAATTCATGGATGATGACTGGG
	3xFLAG-BmVasa RK Reverse (insert)	TGGAGGCACATAGGTTACCGG
	3xFLAG-BmVasa N(Ddx4)-EGFP-C Forward (vector)	ATGGTGAGCAAGGGCGAGG
	3xFLAG-BmVasa N(Ddx4)-EGFP-C Reverse (vector)	GAATTCGATATCCTTGTCATCGTCATCCTTG
	3xFLAG-BmVasa N(Ddx4)-EGFP-C Forward (insert)	AGGATATCGAATTCATGGGAGATGAGGATTGGGAAGC
	3xFLAG-BmVasa N(Ddx4)-EGFP-C Reverse (insert)	GCCCTTGCTCACCATACTTTCGCCGCCTTCAGC
	3xFLAG-BmVasa N(DmVasa)-EGFP-C Forward (vector)	ATGGTGAGCAAGGGCGAGG
	3xFLAG-BmVasa N(DmVasa)-EGFP-C Reverse (vector)	GAATTCGATATCCTTGTCATCGTCATCC
	3xFLAG-BmVasa N(DmVasa)-EGFP-C Forward (insert)	AAGGATATCGAATTCATGTCTGACGACTGGGATGATGAG
	3xFLAG-BmVasa N(DmVasa)-EGFP-C Reverse (insert)	GCCCTTGCTCACCATCTCTCGCTTCCTTTCGACATCTTCG
	3xFLAG-BmVasa N(Ddx4 FA)-EGFP-C Forward (vector)	GAAGGCGGCGAAAGTATGGTGAGCAAGGGCGAGG
	3xFLAG-BmVasa N(Ddx4 FA)-EGFP-C Reverse (vector)	ATCCTCATCTCCCATGAATTCGATATCCTTGTCATCGTCATCC
	3xFLAG-EGFP-BmVasa WT Forward (vector)	GGTTCTATCGAATTCATGGATGATGACTGGG
	3xFLAG-EGFP-BmVasa WT Reverse (vector)	GCTCACCATGGTGAAATCCTTGTCATCGTCATCCTTGTAATC
	3xFLAG-EGFP-BmVasa WT Forward (insert)	TTCACCATGGTGAGCAAGGGC
	3xFLAG-EGFP-BmVasa WT Reverse (insert)	GAATTCGATAGAACCCTTGTACAGCTCGTCCATGC
	3xFLAG-EGFP-BmVasa RK Forward (vector)	ACCTATGTGCCTCCAGAACCAAC
	3xFLAG-EGFP-BmVasa RK Reverse (vector)	GAATTCGATAGAACCCTTGTACAGCTC
	3xFLAG-EGFP-BmVasa RK Forward (insert)	GGTTCTATCGAATTCATGGATGATGACTGGGATGATTCCTG
	3xFLAG-EGFP-BmVasa RK Reverse (insert)	TGGAGGCACATAGGTTACCGG
	3xFLAG-BmVasa R470A Forward (mutagenesis PCR)	GCTGAGGAAGCGTTACAGAATTTT
	3xFLAG-BmVasa R470A Reverse (mutagenesis PCR)	CTCACGTTGCATGCGATCGCCGTG
	3xMyc-BmVasa WT Forward (vector)	TAGACGCGTACCGGTCATCATCAC
	3xMyc-BmVasa WT Reverse (vector)	GAATTCTAAATCCTCCTCACTAAT
	3xMyc-BmVasa WT Forward (insert)	GAGGATTTAGAATTCATGGATGATGACTGGGATGATTCC
	3xMyc-BmVasa WT Reverse (insert)	ACCGGTACGCGTCTACCATTCTTCTTCAGGTTC
	3xMyc-BmVasa ΔN Forward (vector)	TAGACGCGTACCGGTCATCATCAC
	3xMyc-BmVasa ΔN Reverse (vector)	GAATTCTAAATCCTCCTCACTAAT
	3xMyc-BmVasa ΔN Forward (insert)	GAGGATTTAGAATTCACCTATGTGCCTCCAGAACCAACA
	3xMyc-BmVasa ΔN Reverse (insert)	ACCGGTACGCGTCTACCATTCTTCTTCAGGTTC
	3xMyc-BmVasa ΔC Forward (vector)	TAGACGCGTACCGGTCATCATCAC
	3xMyc-BmVasa ΔC Reverse (vector)	GAATTCTAAATCCTCCTCACTAAT
	3xMyc-BmVasa ΔC Forward (insert)	GAGGATTTAGAATTCATGGATGATGACTGGGATGATTCC
	3xMyc-BmVasa ΔC Reverse (insert)	ACCGGTACGCGTCTACTTTAAGAAGTCTGGGACAGATTG
	3xMyc-BmVasa R470A Forward (mutagenesis PCR)	CGTGAGGCAGAGGAAGCGTTACAGAATTTTAAGAG
	3xMyc-BmVasa R470A Reverse (mutagenesis PCR)	TTCCTCTGCCTCACGTTGCATGCGATC
	pET47b-BmVasa WT Forward (vector)	TAGGGGTACCAGGATCCGAATTC
	pET47b-BmVasa WT Reverse (vector)	TCCCAGGGGTCCCTGAAAG
	pET47b-BmVasa WT Forward (insert)	CAGGGACCCCTGGGAATGGATGATGACTGGGATGATTCC
	pET47b-BmVasa WT Reverse (insert)	ATCCTGGTACCCCTACCATTCTTCTTCAGGTTC
	pET47b-BmVasa ΔN Forward (vector)	TAGGGGTACCAGGATCCGAATTC
	pET47b-BmVasa ΔN Reverse (vector)	TCCCAGGGGTCCCTGAAAG
	pET47b-BmVasa ΔN Forward (insert)	CAGGGACCCCTGGGAACCTATGTGCCTCCAGAACCAACA
	pET47b-BmVasa ΔN Reverse (insert)	ATCCTGGTACCCCTACCATTCTTCTTCAGGTTC
	pET47b-BmVasa R470A Forward (mutagenesis PCR)	CGTGAGGCAGAGGAAGCGTTACAGAATTTTAAGAG
	pET47b-BmVasa R470A Reverse (mutagenesis PCR)	TTCCTCTGCCTCACGTTGCATGCGATC
	pET47b-BmVasa E339Q Forward (mutagenesis PCR)	CTGGATCAGGCTGACCGTATGTTAGATATGG
	pET47b-BmVasa E339Q Reverse (mutagenesis PCR)	GTCAGCCTGATCCAGTACGACAAATCTTACACTGC
	pET47b-BmVasa N-IDR Forward (mutagenesis PCR)	AGCCGGTATAGGGGTACCAGGATCCGAATTCTG
	pET47b-BmVasa N-IDR Reverse (mutagenesis PCR)	ACCCCTATACCGGCTTCTTCGTTTCCCCGTTTTC
in vitro ssRNA transcription	50 nt RNA (lacZ) Forwaed	AAAAATTAATACGACTCACTATAGGGATGATAGATCCCGTCGTTTTA
	50 nt RNA (lacZ) Reverse	CCAGGGTTTTCCCAGTCACG
	50 nt RNA (lacZ antisense) Forwaed	ATGATAGATCCCGTCGTTTTACAAC
	50 nt RNA (lacZ antisense) Reverse	AAAAATTAATACGACTCACTATAGGGCCAGGGTTTTCCCAGTCACG
	50 nt RNA (CD) Forwaed	AAAAATTAATACGACTCACTATAGGGATGGCTAAACAAGCAAAGATAG
	50 nt RNA (CD) Reverse	TCAAAAATGGGCATCATTATGTC
	2000 nt RNA (lacZ) Forwaed	AAAAATTAATACGACTCACTATAGGGATGATAGATCCCGTCGTTTTA
	2000 nt RNA (lacZ) Reverse	AGAGGCACTTCACCGCTTGCC
	2000 nt RNA (CD) Forwaed	AAAAATTAATACGACTCACTATAGGGATGGCTAAACAAGCAAAGATAG
	2000 nt RNA (CD) Reverse	ATACCAAAAAATATTTCATAAGGAGTTTTATTCTC
RNAi	siLuc Sense	*CGUACGCGGAAUACUUCGA* TT
	siLuc antisense	*UCGAAGUAUUCCGCGUACG* TT
	siBmVasa Sense	*CAGCACAGCGACUAUGAGA* TT
	siBmVasa antisense	*UCUCAUAGUCGCUGUGCUG* TT
	siSiwi Sense	*CACUCGGAGGAUAUCUCUU* TT
	siSiwi antisense	*AAGAGAUAUCCUCCGAGUG* TT
	siSpn-E Sense	*CAGACACUCGUAUACAUUA* TT
	siSpn-E antisense	*UAAUGUAUACGAGUGUCUG* TT

*: RNA is shown in itallics

Description of Additional Supplementary Files

Supplementary Movie1: Live imaging of EGFP-BmVasa in BmN4 cells. A longer time-frame movie of Fig. 2a. Frame rate: 7 fps. 1 frame: 3 sec. Scale bar: 2 μm.
