# Supplementary material for: Bombyx Vasa sequesters transposon mRNAs in nuage via phase separation requiring RNA binding and self-association
Source: Nat Commun. 2023 Apr 7;14:1942. doi: 10.1038/s41467-023-37634-2 (PMC10081994; doi:10.1038/s41467-023-37634-2)
Supplement: Supplementary file 3 — Description of Additional Supplementary Files [file 41467_2023_37634_MOESM3_ESM.pdf]

Description of Additional Supplementary Files

Supplementary Movie1: Live imaging of EGFP-BmVasa in BmN4 cells. A longer time-frame movie of Fig. 2a. Frame rate: 7 fps. 1 frame: 3 sec. Scale bar: 2 μm.
