# Supplementary material for: Bombyx Vasa sequesters transposon mRNAs in nuage via phase separation requiring RNA binding and self-association
Source: Nat Commun. 2023 Apr 7;14:1942. doi: 10.1038/s41467-023-37634-2 (PMC10081994; doi:10.1038/s41467-023-37634-2)
Supplement: Supplementary file 5 — Reporting Summary [file 41467_2023_37634_MOESM5_ESM.pdf]

nature portfolio

Corresponding author(s): Mikiko C. Siomi

Last updated by author(s): Mar 6, 2023

Reporting Summary

Nature Portfolio wishes to improve the reproducibility of the work that we publish. This form provides structure for consistency and transparency in reporting. For further information on Nature Portfolio policies, see our Editorial Policies and the Editorial Policy Checklist.

Statistics

For all statistical analyses, confirm that the following items are present in the figure legend, table legend, main text, or Methods section.

n/a	Confirmed	
☐	☒	The exact sample size (*n*) for each experimental group/condition, given as a discrete number and unit of measurement
☐	☒	A statement on whether measurements were taken from distinct samples or whether the same sample was measured repeatedly
☐	☒	The statistical test(s) used AND whether they are one- or two-sided<br>*Only common tests should be described solely by name; describe more complex techniques in the Methods section.*
☒	☐	A description of all covariates tested
☒	☐	A description of any assumptions or corrections, such as tests of normality and adjustment for multiple comparisons
☐	☒	A full description of the statistical parameters including central tendency (e.g. means) or other basic estimates (e.g. regression coefficient) AND variation (e.g. standard deviation) or associated estimates of uncertainty (e.g. confidence intervals)
☐	☒	For null hypothesis testing, the test statistic (e.g. *F*, *t*, *r*) with confidence intervals, effect sizes, degrees of freedom and *P* value noted<br>*Give P values as exact values whenever suitable.*
☒	☐	For Bayesian analysis, information on the choice of priors and Markov chain Monte Carlo settings
☒	☐	For hierarchical and complex designs, identification of the appropriate level for tests and full reporting of outcomes
☐	☒	Estimates of effect sizes (e.g. Cohen's *d*, Pearson's *r*), indicating how they were calculated

Our web collection on statistics for biologists contains articles on many of the points above.

Software and code

Policy information about availability of computer code

Data collection	Chemiluminescence detection was performed by ChemiDoc XRS Plus System (Bio-Rad). Autoradiography was performed by Typhoon FLA 9500 (Cytiva). Deep sequencing was performed with MiSeq (Illumina) and NovaSeq (Illumina).
Data analysis	Python 3.7.6, Numpy 1.18.1, Pandas 1.0.1, Matplotlib 3.1.3, Scipy 1.4.1, Fiji 2.1.0, EMBOSS Needle (https://www.ebi.ac.uk/Tools/psa/emboss_needle/), Cutadapt (version 4.0), FASTX-Toolkit (version 0.0.14), STAR (version 2.7.9a), Bowtie (version 1.3.1), Rcorrector (version 1.0.4), TranscriptomeAssemblyTools (https://github.com/harvardinformatics/TranscriptomeAssemblyTools), TrimGalore (version 0.6.6), SAMtools (version 1.15), and R (version 4.2.0) were used for data analysis.

For manuscripts utilizing custom algorithms or software that are central to the research but not yet described in published literature, software must be made available to editors and reviewers. We strongly encourage code deposition in a community repository (e.g. GitHub). See the Nature Portfolio guidelines for submitting code & software for further information.

Data

Policy information about availability of data

All manuscripts must include a data availability statement. This statement should provide the following information, where applicable:

- Accession codes, unique identifiers, or web links for publicly available datasets
- A description of any restrictions on data availability
- For clinical datasets or third party data, please ensure that the statement adheres to our policy

Source data are provided with this paper. The data supporting the findings of this study are available from the corresponding author upon reasonable request. The

FAST-iCLIP and total RNA sequencing data generated in this study have been deposited in Gene Expression Omnibus under accession code GSE213917 [https://www.ncbi.nlm.nih.gov/geo/query/acc.cgi?acc=GSE213917]. The silkworm genome databases used in this study (KAIKObase database and Silkbase database) are available under the following links: [https://sgp.dna.affrc.go.jp/KAIKObase/, https://silkbase.ab.a.u-tokyo.ac.jp/cgi-bin/index.cgi].

Human research participants

Policy information about studies involving human research participants and Sex and Gender in Research.

Reporting on sex and gender	This study is not related to human research.
Population characteristics	This study is not related to human research.
Recruitment	This study is not related to human research.
Ethics oversight	This study is not related to human research.

Note that full information on the approval of the study protocol must also be provided in the manuscript.

Field-specific reporting

Please select the one below that is the best fit for your research. If you are not sure, read the appropriate sections before making your selection.

- [x] Life sciences
- [ ] Behavioural & social sciences
- [ ] Ecological, evolutionary & environmental sciences

For a reference copy of the document with all sections, see nature.com/documents/nr-reporting-summary-flat.pdf

Life sciences study design

All studies must disclose on these points even when the disclosure is negative.

Sample size	No statistical methods were used to predetermine sample size. Sample sizes were estimated empirically on the basis of pilot experiments and previously performed experiments with similar setup to provide sufficient sample sizes for statistical analysis.
Data exclusions	No data was removed from the analysis.
Replication	Immunoprecipitation of Supplementary Fig. 1e was performed over six independent experiments, and other immunoprecipitation, rescue assay, CLIP, immunofluorescence, live cell imaging, in vitro droplet formation assay, electrophoretic mobility shift assay, FAST-iCLIP, and total RNA sequencing were performed over two independent experiments.
Randomization	This study does not involve animal experiments. Cells were cultured in flasks or dishes and when sufficient cultured cells for the each specific assay were available flasks or dishes were randomly chosen for transfection or knockdown.
Blinding	Experiments performed in this article were not blinded and this is consistent with what is published in the field.

Reporting for specific materials, systems and methods

We require information from authors about some types of materials, experimental systems and methods used in many studies. Here, indicate whether each material, system or method listed is relevant to your study. If you are not sure if a list item applies to your research, read the appropriate section before selecting a response.

Materials & experimental systems

n/a	Involved in the study
[ ]	[x] Antibodies
[ ]	[x] Eukaryotic cell lines
[x]	[ ] Palaeontology and archaeology
[x]	[ ] Animals and other organisms
[x]	[ ] Clinical data
[x]	[ ] Dual use research of concern

Methods

n/a	Involved in the study
[x]	[ ] ChIP-seq
[x]	[ ] Flow cytometry
[x]	[ ] MRI-based neuroimaging

Antibodies

Antibodies used	[Primary antibodies]

Anti-BmVasa and Ago3 monoclonal antibodies were produced from immunized mice, respectively (Nishida et al., 2015, Cell Reports 10, 193-203).
Anti-Flag M2 (catalog number: F3165, Sigma), anti-Flag produced in rabbit (catalog number: F7425, Sigma), anti-Myc (9E10, Developmental Studies Hybridoma Bank) and anti-β-Tubulin (E7, Developmental Studies Hybridoma Bank) monoclonal antibodies were purchased.
For western blotting, each antibody was used at the following dilution [anti-BmVasa (1:20 (supernatant of hybridoma cells)), Flag (1:5000), Myc (1:5000), β-Tubulin (1:1000)].
For immunofluorescence, each antibody was used at the following dilution [anti-Ago3 (1:500), Flag (1:1000)].
[Secondary antibodies]
Peroxidase-conjugated anti-mouse IgG antibody (1:5,000 dilution; catalog number: 55558, Cappel)
TrueBlot ULTRA: Anti-Ig HRP, Mouse (Rat) (1:1,000 dilution; catalog number: eB144, ROCKLAND)
Goat anti-Mouse IgG1 Cross-Adsorbed Secondary Antibody, Alexa Fluor™ 488 (1:1,000 dilution; catalog number: A-21121, Invitrogen)
Goat anti-Mouse IgG2a Cross-Adsorbed Secondary Antibody, Alexa Fluor™ 555 (1:1,000 dilution; catalog number:A-21137, Invitrogen)

Validation

[Primary antibodies]
Anti-BmVasa and Ago3 monoclonal antibodies were validated by Western blotting of a knockdown lysate of BmN4 in a previous studies, respectively (Nishida et al., 2015, Cell Reports 10, 193-203).
For the following purchased antibodies, see manufacturer information:
Anti-Flag M2 (https://www.sigmaaldrich.com/JP/ja/product/sigma/f3165)
Anti-Myc (https://dshb.biology.uiowa.edu/9E-10)
Anti-β-Tubulin (https://dshb.biology.uiowa.edu/E7_2)
[Secondary antibodies]
Peroxidase-conjugated anti-mouse IgG antibody (https://www.mpbio.com/jp/0855558-peroxidase-conjugated-sheep-igg-fraction-to-mouse-igg-no-cross-to-human)
TrueBlot ULTRA: Anti-Ig HRP, Mouse (Rat) (https://www.rockland.com/categories/trueblot/mouse-trueblot-ultra-anti-mouse-ig-hrp-18-8817-30/)
Goat anti-Mouse IgG1 Cross-Adsorbed Secondary Antibody, Alexa Fluor™ 488 (https://www.thermofisher.com/antibody/product/Goat-anti-Mouse-IgG1-Cross-Adsorbed-Secondary-Antibody-Polyclonal/A-21121)
Goat anti-Mouse IgG2a Cross-Adsorbed Secondary Antibody, Alexa Fluor™ 555 (https://www.thermofisher.com/antibody/product/Goat-anti-Mouse-IgG2a-Cross-Adsorbed-Secondary-Antibody-Polyclonal/A-21137)

Eukaryotic cell lines

Policy information about cell lines and Sex and Gender in Research

Cell line source(s)	BmN4 cells were gifted from National Institute of Agrobiological Sciences (NIAS).
Authentication	BmN4 cell have not been authenticated.
Mycoplasma contamination	BmN4 cells were not tested for mycoplasma contamination.
Commonly misidentified lines (See ICLAC register)	BmN4 cells are not misidentified cell lines.
